# 12-week curcumin supplementation may relieve postexercise muscle fatigue in adolescent athletes

**DOI:** 10.3389/fnut.2022.1078108

**Published:** 2023-01-04

**Authors:** Kai-Yuan Bai, Gin-Hua Liu, Chun-Hao Fan, Liang-Tseng Kuo, Wei-Hsiu Hsu, Pei-An Yu, Chi-Lung Chen

**Affiliations:** ^1^Department of Orthopedic Surgery, Chang Gung Memorial Hospital, Chiayi, Taiwan; ^2^Department of Nutrition, Chang Gung Memorial Hospital, Chiayi, Taiwan; ^3^Sports Medicine Center, Chang Gung Memorial Hospital, Chiayi, Taiwan; ^4^Division of Sports Medicine, Department of Orthopedic Surgery, Chang Gung Memorial Hospital, Chiayi, Taiwan; ^5^School of Medicine, Chang Gung University, Taoyuan, Taiwan

**Keywords:** natural polyphenols, curcumin, muscle-damaging exercise, anti-inflammatory, antioxidants, athletic performance

## Abstract

**Introduction:**

High-intensity exercise causes oxidative stress, muscle soreness, and muscle fatigue, leading to reduced exercise performance. Curcumin possesses antioxidative and anti-inflammatory properties and thus alleviates postexercise damage. Therefore, this study evaluated the effect of curcumin on athletes’ postexercise recovery.

**Methods:**

A non-randomized prospective cohort investigation was done. We recruited middle and high school athletes engaged in wrestling, soccer, and soft tennis. During the 12-week daily exercise training, the participants were assigned to receive curcumin supplementation (curcumin group) or not (control group). Body composition, exercise performance, inflammatory factors, muscle fatigue, and muscle soreness were recorded at the baseline and end of the study. We used the Mann–Whitney U test to compare the participants’ demographics, such as age, height, weight, and training years. The Wilcoxon test was used to compare the differences between the groups before and after curcumin supplementation.

**Results:**

Of 28 participants (21 men and 7 women, with a mean age of 17 years), 13 were in the curcumin group and 15 in the control group. A significant decrease in muscle fatigue and muscle soreness scores was observed in the curcumin group after 12 weeks. Moreover, a significant decrease in the 8-hydroxy-2 deoxyguanosine level and a significant increase in basic metabolic rate and fat-free mass were observed in the curcumin group.

**Conclusion:**

Curcumin can reduce muscle fatigue and soreness after exercise, indicating its potential to alleviate postexercise damage. It could be considered to cooperate with nutritional supplements in regular training in adolescent athletes.

## Introduction

Regular exercise has many health benefits (1); however, intense physical activity, especially eccentric muscle contractions, causes exercise-induced muscle damage (EIMD) and delayed onset muscle soreness (DOMS), which can affect athletic performance (2). Moreover, EIMD-induced inflammatory response may lead to several negative effects, including limited strength, decreased range of motion (ROM), and increased circulating levels of muscle damage markers (2). Decreased muscle strength in EIMD stems from sarcomere damage, impaired neuromuscular function, reduced ROM, muscle pain, and swelling (3) can persist for 3–5 days (4). Moreover, EIMD-induced inflammatory responses activate transcription factors, produce extracellular reactive oxygen species (ROS), and increase serum levels of proinflammatory cytokines, such as C-reactive proteins and interleukins [interleukin-1 (IL-1) and tumor necrosis factor-α (TNF-α)]. Besides, EIMD is also caused by free radicals, which lead to oxidative stress and reactive nitrogen species and resultant nitrosative stress (5, 6). Because of the hyperpermeability of cell membranes, muscle proteins, including creatine kinase (CK) and lactate dehydrogenase (LDH), are increasingly released from muscle cells into systemic circulation. These proteins negatively affect muscle strength (7) and are, therefore, used as biomarkers of muscle damage (8).

Because of their negative impact on athletic performance, muscle damage, and inflammatory responses must be controlled for rapid recovery, especially in athletes and those undergoing intense exercise regimens. Nonsteroidal anti-inflammatory drugs (NSAIDs) are commonly used to treat DOMS (9); however, their effectiveness after EIMD is limited (10). Because of the detrimental effects of NSAIDs on soft tissue healing and the central nervous system (11), less harmful natural substances with anti-inflammatory potential must be explored.

Curcumin [1,7-bis (4-hydroxy-3-methoxyphenyl) 1,6-heptadiene-3,5-dione], the main natural bioactive polyphenol of turmeric (2 to 5% by weight), possesses anti-inflammatory and antioxidant properties (12, 13) and may be used to increase exercise performance (14). Curcumin can inhibit the formation of cyclooxygenase and lipoxygenase and can indirectly reduce free radical formation (15). In addition, curcumin can inhibit intracellular ROS production and increase the levels of antioxidant enzymes, such as vitamin E and coenzyme Q10 (15, 16). Curcumin reduces inflammation by inhibiting inducible nitric oxide synthase production and nitric oxide release and by reducing the activity of nuclear factor kappa B and cytokines, such as IL-1 β, TNF-α, and IL-6 β (16). Moreover, curcumin can inhibit STAT3 activation, another key inflammatory pathway (17, 18).

The short-term effects of curcumin on exercise were approved by animal studies (15, 19). Recent systematic reviews have supported the clinical efficacy of curcumin in treating oxidative stress, EIMD, and DOMS (2, 20, 21). Several studies investigated the efficacy of curcumin on postexercise muscle damage (22–27). However, most of them focused on the untraining period (24–27) or had a short-term study period (< 1 week) (24–27). Therefore, the long-term applicability of curcumin during the training period was uncertain, especially during the regular training status. Although some studies focused on the long-term supplement of curcumin during the training period (22, 23), the participants were all young adults (mean age > 20 years). Therefore, the effect of curcumin on adolescent athletes’ performance and muscle damage remains uncertain. Thus, we explored the efficacy of curcumin supplements in adolescent athletes in this study. We hypothesized that curcumin supplementation can effectively reduce oxidative stress, inflammatory index, and muscle damage and improve sports performance after training in adolescent athletes.

## Materials and methods

### Participants

This study enrolled middle and high school athletes receiving sub-special training, such as wrestling, soft tennis, and soccer. Participants who received regular training for more than 20 h per week for more than 1 year were included. Participants who were regularly taking supplements, such as turmeric, vitamins, and zinc, were excluded. Participants who were allergic to curcumin or felt uncomfortable with curcumin supplements were also excluded. We also excluded athletes who cannot receive regular training due to sports injuries to minimize bias from training levels. After enrollment, the participants were provided the option of joining either the curcumin group or the control group. The participants in the curcumin group received the curcumin supplement, whereas those in the control group did not. All participants performed exercise training in the Chiayi County Yung Ching Senior High School and recorded the amount of daily exercise training in an exercise training log. We explained the study project to the participants and their guardians and obtained a signed informed consent form. This study conformed to the tenets of the Declaration of Helsinki for medical research involving human subjects and was approved by the Institutional Review Board of Chang Gung Memorial Hospital (IRB No. 202100069A3C601).

The participants’ demographics, including age (years), height (cm), weight (kg), sports training experience (years), details of training undergone, nutritional supplement status, and medical history, were recorded. In addition, all participants underwent body composition, blood, urine, fitness, muscle fatigue, and muscle soreness assessments at the baseline and end of this 12-week study. No exercise training was scheduled on the test day.

### Nutritional supplements

The participants in the curcumin group received a Chinese curcumin supplement named Jiang Huang Powder Ko Da (KO DA Pharmaceutical Co., Ltd., Taoyuan, Taiwan) after being evaluated by a Chinese medical practitioner. Each pack contains 500 mg of Jiang Huang Powder, which is made by 300 mg of curcumin, 190 mg of starch, and 10 mg of sodium carboxymethyl cellulose. The participants received a 1.5-g sachet a day for 90 consecutive days; therefore, the curcumin intake of each participant was 1,200 mg per day.

### Body composition

We measured the participants’ body composition by using a body composition analyzer (InBody 720, Biospace, Seoul, Republic of Korea), which simultaneously records body weight, fat-free mass, body fat mass, and basal metabolic rate (BMR) (28).

### Blood and urine analysis

We determined the serum TNF-α and CK levels to monitor the inflammatory index and muscle damage. In addition, we assessed the participants’ oxidative stress index by determining the levels of 8-hydroxy-2 deoxyguanosine (8-OHdG) and malondialdehyde (MDA) in urine.

Spot urine samples were collected and stored below –80°C until the analysis. Urinary MDA was quantified using a thiobarbituric acid reactive substances assay kit (Oxford Biomedical Research Inc., Rochester Hills, MI, USA. Product codes: FR40). Urinary 8-OHdG was quantified using an ultraperformance liquid chromatography–tandem mass spectrometer (Waters Xevo TQ-XS, Milford, MA, USA. Product codes: WAT058951). Urinary creatinine (CRE) concentration was used for urinary MDA and 8-OHdG concentration adjustments.

For determining the levels of CK and TNF-α, 5 mL of blood samples were collected into a serum separation tube through cubital fossa venipuncture in the dominant arm. Serum TNF-α was measured using chemiluminescent immunoassay on a Siemens IMMULITE 1000 system (Siemens Healthcare Diagnostics Inc., Camberley, Surrey, UK. Product Codes: LKNF1). In addition, serum CK activity was determined using the enzymatic rata method with a Hitachi LST008 (Hitachi, Japan) and a test kit (Cica LaboFit CK, Japan).

The concentration of CK, TNF-α, and MDA was determined at the Department of Laboratory Medicine, Chang Gung Memorial Hospital, Chiayi. The 8-OHdG quantification was performed at the Department of Laboratory Medicine, Chang Gung Memorial Hospital, Linkou. Both laboratories are accredited to ISO 15189 by Taiwan Accreditation Foundation.

### Fitness assessment

The participants’ fitness level was evaluated using the HELMAS physical fitness management system (O2RUN CO., LTD., Seoul, Republic of Korea) at our institution. Several dimensions of health-related fitness, including muscular strength (grip and back strength), balance (closed-eyes foot balance), flexibility (sitting trunk flexion and trunk extension), muscle endurance (sit-ups), power (vertical jump), and agility (reaction times and side steps), were evaluated. The participants warmed up for 5 min before the test. To minimize the influence of fatigue, the participants rested for 1 min between each test. Each test was performed twice (with a minimum interval of 30 s), and the higher performance was selected as a measurement of individual physical fitness (29).

### Muscle fatigue and muscle soreness assessment

We used the visual analog scale to assess the participants’ muscle fatigue and muscle soreness status, with 0 and 10 cm denoting no fatigue or soreness and maximum fatigue or soreness, respectively (30). The participants filled out the assessment form daily after the training.

### Effect size

The effect size (Cohen’s *d*) was 1.13, as determined using G*Power 3.1.9.7 (Heinrich Heine University, Düsseldorf, Germany). The input parameters used for the two-tailed *t* test were alpha = 0.05, power = 0.8, the sample size of the control group was 15, and the sample size of the curcumin group was 13.

### Statistical analysis

All data were analyzed using SPSS 26.0 (SPSS Inc., Chicago, IL, USA). Means (standard deviation) were calculated for the participants’ parameters. The Mann–Whitney *U* test was used to compare the parameters between the groups. The Wilcoxon test was used to compare the baseline and 12-week parameters in the same group. In addition, the change in each item was calculated using the following formula:


$$Change\;(\%)=\left(Test_{12\,t\,h\,w\,e\,e\,k}-Test_{b\,a\,s\,e\,l\,i\,n\,e}/Test_{b\,a\,s\,e\,l\,i\,n\,e}\right)\times 100\%$$

The percentage change between baseline and 12th week was compared in both groups. We used the chi-square test to compare the percentage change in parameters across the study period between the control and curcumin groups; *p* < 0.05 was considered significant.

## Results

**FIGURE 1:**
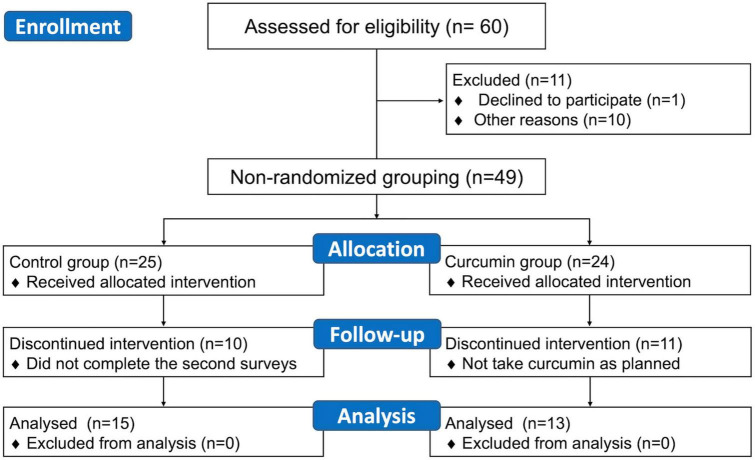
Study schematic.

This study initially enrolled 60 participants. After 11 participants were excluded, 49 remained. Of them, 25 and 24 participants were in the control and curcumin groups, respectively. Because of an incomplete second survey, 10 participants from the control group were excluded. Moreover, 11 participants in the curcumin group were excluded due to nonadherence to the curcumin regimen. Finally, 28 participants, with 15 (13 male and 2 female) in the control group and 13 (8 male and 5 female) in the curcumin group, were included in the study (Figure 1).

### Body composition analysis

Female athletes had a lower fat-free mass and BMR than the males in both groups (Supplementary Table 1). In the control group, no significant change in body composition was observed after 12 weeks. However, in the curcumin group, a significant increase in fat-free mass (*p* = 0.003) and BMR (*p* = 0.001) was observed after 12 weeks (Table 1). Furthermore, the significant increase in BMR was constant through box gender, whereas increase of fat-free mass was only significant in males (Supplementary Table 1).

**TABLE 1:** Demographics and body composition data of the participants.

	Control (*N* = 15)				Curcumin (*N* = 13)			
Gender, F: M	2: 13				5: 8			
Sports type, W: S: ST	3: 9: 3				2: 9: 2			
Age (years)	17 ± 1				17 ± 1			
Height (cm)	168 ± 6				165 ± 7			
	**Baseline**		**12 weeks follow up**		**Baseline**		**12 weeks follow up**	
	**Mean**	**± SD**	**Mean**	**± SD**	**Mean**	**± SD**	**Mean**	**± SD**
Weight (kg)	61	± 9	61	± 7	62	± 7	63	± 7
BMI (kg/m^2^)	22	± 3	22	± 2	23	± 3	23	± 2
Body fat mass (kg)	9	± 6	9	± 5	13	± 6	12	± 4
Fat free mass (kg)	51	± 7	52	± 7	49	± 7	51	± 8^†^
BMR (kcal)	1478	± 142	1484	± 153	1433	± 153	1464	± 160^†^

^†^*p* < 0.05 between baseline and 12 weeks. BMI, body mass index; BMR, basal metabolic rate; F, female; M, male; S, soccer; ST, soft tennis; W, wrestling.

### Blood and urine analysis

We assessed the levels of CK, MDA, 8-OHdG, and TNF-α (Table 2). In the curcumin group, a significant reduction in the urinary level of 8-OHdG was observed after 12 weeks, with a decrease in mean value from 4.79 to 3.86 ng/mg CRE (*p* < 0.036). However, no significant changes were noted in the levels of CK, MDA, and TNF-α (all *p* > 0.05). Furthermore, the subgroup analysis according to gender did not find the significant difference of change as above (Supplementary Table 2).

**TABLE 2:** Inflammatory markers, fitness, and muscle fatigue at baseline and the end of study (12 week).

	Control (*N* = 15)		Curcumin (*N* = 13)	
	**Baseline**	**12 weeks follow up**	**Baseline**	**12 weeks follow up**
	**Mean ± SD**	**Mean ± SD**	**Mean ± SD**	**Mean ± SD**
**Inflammatory markers**				
CK (U/L)	446 ± 375	536 ± 669	277 ± 113	290 ± 165
MDA (μmol/g CRE)	1.45 ± 0.57	1.83 ± 0.71	1.60 ± 1.53	1.22 ± 1.07
8-OHdG (ng/mg CRE)	4.10 ± 1.23	4.21 ± 1.55	4.79 ± 1.66	3.86 ± 1.12†
TNF-α (pg/ml)	7.23 ± 1.80	6.70 ± 1.67	6.75 ± 1.36	6.91 ± 1.01
**Fitness**				
Sit up (times)	24 ± 5	24 ± 5	21 ± 3*	22 ± 2
Back strength (kg)	109 ± 23	123 ± 27†	109 ± 22	123 ± 23†
Grip strength (kg)	39 ± 7	40 ± 7	38 ± 10	37 ± 9
Vertical jump (cm)	43 ± 9	47 ± 7†	39 ± 10	42 ± 8†
Side step (times)	35 ± 7	36 ± 8	37 ± 8	34 ± 6
Reaction time (ms)	261 ± 58	302 ± 69	330 ± 151	256 ± 51
Close eyes foot balance (s)	49 ± 41	58 ± 53	98 ± 122	72 ± 56
Sitting trunk flexion (cm)	16 ± 5	16 ± 7	16 ± 7	15 ± 6
Trunk extension (cm)	46 ± 4	49 ± 6	48 ± 6	50 ± 6
**Muscle fatigue**				
Muscle fatigue score	7 ± 1	7 ± 1	6 ± 1	4 ± 2*†
Muscle soreness score	7 ± 1	7 ± 2	7 ± 2	4 ± 2*†

*p < 0.05 between the control and curcumin groups. ^†^*p* < 0.05 between baseline and 12 weeks. 8-OHdG, 8-hydroxy-2 deoxyguanosine; CK, creatine kinase; CRE, creatinine; MDA, malondialdehyde; TNF-α, tumor necrosis factor-α; SD, standard deviation.

### Fitness and muscle fatigue assessment

In the curcumin group, the muscle fatigue score and muscle soreness score decreased significantly from 6 ± 1 to 7 ± 2, respectively, at baseline to 4 ± 2 and 4 ± 2, respectively, after 12 weeks of curcumin supplementation (all *p* = 0.005; Table 2). These significant changes were still noted in males, but not in females (Supplementary Table 2). A significant difference in reaction time, muscle fatigue score, and muscle soreness score was observed between the groups. The mean percentage change in reaction time of the control and curcumin groups was 21 and -14%, respectively (both *p* < 0.05; Table 3). Furthermore, significant differences in percentage change in muscle fatigue score and muscle soreness score were observed between the groups (*p* = 0.007 and *p* = 0.001, respectively, Table 3). Similarly, these significant changes were still noted in males, but not in females (Supplementary Table 3).

**TABLE 3:** Percentage change of body composition, inflammatory markers, fitness, and muscle fatigue between baseline and the end of study (12 weeks).

	Control (*N* = 15)	Curcumin (*N* = 13)	
	**Mean ± SD**	**Mean ± SD**	***p***
**Weight and body composition**			
Weight	1 ± 4	2 ± 4	0.565
Body fat mass	8 ± 27	1 ± 17	0.761
Fat-free mass	1 ± 5	3 ± 2	0.093
BMR	0.4 ± 3	2 ± 1	0.076
**Inflammatory markers**			
CK	19 ± 104	9 ± 51	0.662
MDA	32 ± 64	22 ± 149	0.439
8-OHdG	3 ± 22	-14 ± 27	0.050
TNF-α	-7 ± 26	6 ± 23	0.237
**Fitness**			
Sit up	-1 ± 20	6 ± 15	0.253
Back strength	12 ± 14	14 ± 16	0.846
Grip strength	4 ± 14	1 ± 13	0.698
Vertical jump	9 ± 11	11 ± 18	0.560
Side step	5 ± 22	-4 ± 24	0.382
Reaction time	21 ± 38	-14 ± 25	0.017*
Close eyes foot balance	89 ± 219	8 ± 93	0.357
Sitting trunk flexion	-6 ± 24	6 ± 38	0.923
Trunk extension	6 ± 13	6 ± 14	0.898
**Muscle fatigue**			
Muscle fatigue score	2 ± 21	-29 ± 29	0.007*
Muscle soreness score	-5 ± 28	-36 ± 29	0.001*

*p < 0.05 between the control and curcumin groups.

### Comparison of percentage change between groups

**FIGURE 2:**
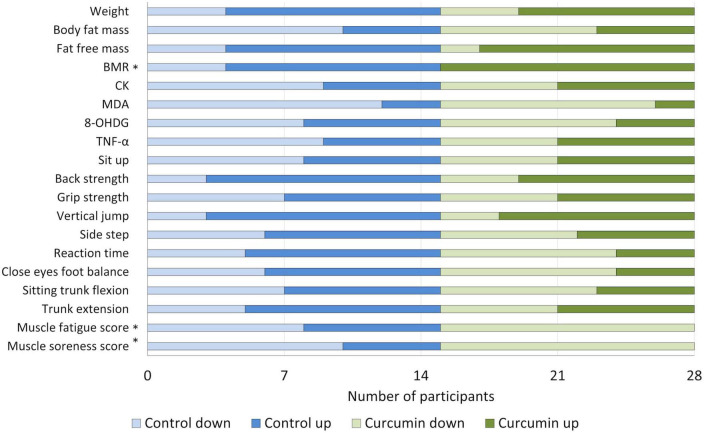
Comparison of change trends of parameters between the two groups. Asterisk means statistically significant difference between the control and curcumin groups (Chi-square test: BMR, *p* = 0.044; muscle fatigue score, *p* = 0.004; muscle soreness score, *p* = 0.022). 8-OHdG, 8-hydroxy-2 deoxyguanosine; BMR, basic metabolic rate; CK, creatinine kinase; MDA, malondialdehyde; TNF-α, tumor necrosis factor-α.

We used the chi-square test to compare the percentage change in parameters before and after supplementation between the control and curcumin groups (Figure 2). A significant difference in percentage change (decrease) in BMR, muscle fatigue score, and muscle soreness score (*p* = 0.044, 0.004, and 0.022, respectively) was observed between the groups.

## Discussion

This study examined changes in body composition, inflammation, exercise performance, and muscle fatigue in adolescent athletes after curcumin supplementation. In the curcumin group, a significant decrease in the 8-OHdG level, muscle fatigue score, and muscle soreness score was observed compared with the baseline values. A significant difference in reaction time, muscle fatigue score, and muscle soreness score was observed between the curcumin and control groups. Moreover, a significant difference in percentage change in BMR, muscle fatigue score, and muscle soreness score was observed between the groups. These findings imply that curcumin supplementation not only reduces inflammation but also reduces postexercise muscle fatigue and soreness in adolescent athletes. Therefore, curcumin supplements can alleviate muscle damage after intensive exercise, and should be considered to be incorporated into the nutrition supplement during regular training in adolescent athletes.

Many studies have indicated the antioxidant and anti-inflammatory properties of curcumin (2, 14, 31). Inflammatory mediators are released during immune system activation after EIMD, and curcumin may lower inflammatory mediator levels through the regulation of signaling pathways (2). Nicol et al. and Tanabe et al. discovered that curcumin cannot inhibit TNF-α, which is consistent with our findings (26, 32). However, McFarlin et al. discovered that curcumin supplementation significantly reduced TNF-α levels after leg press exercise (27). Some studies have indicated that curcumin reduces postexercise CK rise (20, 26), a result not observed in this study. Roohi et al. discovered that curcumin supplementation after a 14-km run significantly reduced MDA levels (33). These results indicated that curcumin supplementation may affect antioxidant markers differently. Curcumin supplementation significantly reduced only 8-OHdG levels in our study. Although curcumin cannot reduce the levels of all oxidative metabolites, it can reduce some oxidative stress caused by exercise, thereby increasing the overall antioxidant capacity (24, 34).

Jäger et al. discovered that curcumin slowed muscle strength loss after muscle-damaging exercise (22). Nicol et al. discovered that curcumin may improve jumping performance in the days following muscle damage (32). We evaluated several parameters of athletic performance; of them, only reaction time exhibited a significant difference after 12 weeks. The difference may arise from the assessment frequency. The previous studies [20, 30] which repeated the assessments several times after exercise, observed that the curcumin group had a less reduction in athletic performance than the control group. Our study only made an assessment once after the curcumin supplement. In future research, serial assessments may be needed to check the curcumin efficacy in athletic performance in adolescents.

Several studies have analyzed the effect of curcumin on postexercise recovery (20, 23, 25–27, 35) and described that curcumin supplementation could relieve postexercise muscle soreness (20, 23, 25). However, other studies have reported contradictory results (26, 27). Tanabe et al. (26) and Mcfarlin et al. (27) discovered that pre-exercise and postexercise curcumin supplementation did not relieve muscle soreness. However, we discovered that curcumin supplementation alleviated postexercise fatigue and muscle soreness. These contradictory results may be because some studies have used a single dose of curcumin before and after exercise or have used a shorter supplementation period; however, we used a daily dose of curcumin over 12 weeks. Compared with a single dose before and after exercise, regular and quantitative daily doses may help achieve a stable blood concentration and exert a superior effect.

A study focused on changes in certain muscle groups after performing a single course of specific movements, such as concentric or eccentric contraction of a specific joint, treadmill walking, leg press, and running (35). Moreover, these studies were performed for a short period (within 3 days). However, in the present study, the exercises focused on the whole body. Moreover, the study period was longer (12 weeks), leading to an improved understanding of how curcumin affects overall athletic performance and postexercise recovery after a prolonged training session.

### Strengths and limitations

In this study, some strengths may be highlighted, such as the first study investigating the effect of curcumin on postexercise recovery in adolescent athletes and a more extended intervention period (12 weeks) than ever. However, this study has several limitations. First, because of not a randomized, double-blind study with limited samples, inherent biases may exist. Although the baseline characteristics of the participants were similar, the participants were provided the option of joining either the curcumin group or the control group, thereby leading to a selection bias. Moreover, the participants and assessors were not blinded to the treatment, contributing to performance bias and detection bias. Limited samples jeopardized the interpretation of subgroup analyses by gender. These biases reduced the certainty of the results. Second, we only checked some inflammatory markers; therefore, the effect of curcumin on other inflammatory markers, such as IL-6, IL-8, nuclear factor kappa B, LDH, and lactate, in adolescent athletes could not be determined. Third, we did not develop a standardized protocol for sports training but continued the training the players were already undergoing. Although the inclusion of different sports-based exercises could have influenced the interpretation of the results, we used the exercise training log that recorded parameters, including the session rating of perceived exertion, to monitor the training load, which may somewhat limit this bias (36). Fourth, we did not standardize the participants’ nutritional intake or record each participant’s diet. Some diets containing natural curcumin, such as yellow mustard and curry, might potentially interfere with the results of this study. To eliminate the possible bias from unbalanced nutritional intake and diet, an athlete should be requested to design a diet tailored to their situation and record them in future investigations.

## Conclusion

Regular curcumin supplementation during exercise can relieve the associated muscle soreness and fatigue. Although the effect of curcumin supplementation on exercise performance may vary from person to person, it could be considered to cooperate into nutritional supplements in regular training in adolescent athletes.

## Data availability statement

The raw data supporting the conclusions of this article will be made available by the authors, without undue reservation.

## Ethics statement

The studies involving human participants were reviewed and approved by Institutional Review Board of Chang Gung Memorial Hospital (IRB No. 202100069A3C601). Written informed consent to participate in this study was provided by the participants or their legal guardian/next of kin.

## Author contributions

G-HL: conceptualization and methodology. K-YB: funding acquisition and writing—original draft preparation. C-HF: project administration, investigation, data curation, and formal analysis. L-TK and W-HH: methodology and writing—review and editing. W-HH: conceptualization and supervision. All authors have read and agreed to the published version of the manuscript.

## References

[1] Warburton DER Bredin SSD. Health benefits of physical activity: a systematic review of current systematic reviews. *Curr Opin Cardiol.* (2017) 32:541–56. 10.1097/HCO.0000000000000437 28708630

[2] Fernández-Lázaro D Mielgo-Ayuso J Seco Calvo J Córdova Martínez A Caballero García A Fernandez-Lazaro CI. Modulation of exercise-induced muscle damage, inflammation, and oxidative markers by curcumin supplementation in a physically active population: a systematic review. *Nutrients.* (2020) 12:501. 10.3390/nu12020501 32075287 PMC7071279

[3] Marcora SM Bosio A. Effect of exercise-induced muscle damage on endurance running performance in humans. *Scand J Med Sci Sports.* (2007) 17:662–71. 10.1111/j.1600-0838.2006.00627.x 17346288

[4] Fochi AG Damas F Berton R Alvarez I Miquelini M Salvini TF Greater eccentric exercise-induced muscle damage by large versus small range of motion with the same end-point. *Biol Sport.* (2016) 33:285–9. 10.5604/20831862.1208480 27601784 PMC4993145

[5] Dane S Taysi S Gul M Akcay F Gunal A. Acute exercise induced oxidative stress is prevented in erythrocytes of male long distance athletes. *Biol Sport.* (2008) 25:115.

[6] Taysi S Tascan AS Ugur MG Demir M. Radicals, oxidative/nitrosative stress and preeclampsia. *Mini Rev Med Chem.* (2019) 19:178–93. 10.2174/1389557518666181015151350 30324879

[7] Sewright KA Hubal MJ Kearns AMY Holbrook MT Clarkson PM. Sex differences in response to maximal eccentric exercise. *Med Sci Sports Exerc.* (2008) 40:242–51. 10.1249/mss.0b013e31815aedda 18202579

[8] Brancaccio P Lippi G Maffulli N. Biochemical markers of muscular damage. *Clin Chem Lab Med.* (2010) 48:757–67. 10.1515/CCLM.2010.179 20518645

[9] Beiner JM Jokl P Cholewicki J Panjabi MM. The effect of anabolic steroids and corticosteroids on healing of muscle contusion injury. *Am J Sports Med.* (1999) 27:2–9. 10.1177/03635465990270011101 9934411

[10] Schoenfeld BJ. The use of nonsteroidal anti-inflammatory drugs for exercise-induced muscle damage: implications for skeletal muscle development. *Sports Med.* (2012) 42:1017–28. 10.1007/BF03262309 23013520

[11] Auriel E Regev K Korczyn AD. Nonsteroidal anti-inflammatory drugs exposure and the central nervous system. *Handb Clin Neurol.* (2014) 119:577–84. 10.1016/B978-0-7020-4086-3.00038-2 24365321

[12] Chattopadhyay I Biswas K Bandyopadhyay U Banerjee RK. Turmeric and curcumin: biological actions and medicinal applications. *Curr Sci.* (2004) 87:44–53.

[13] Maheshwari RK Singh AK Gaddipati J Srimal RC. Multiple biological activities of curcumin: a short review. *Life Sciences.* (2006) 78:2081–7. 10.1016/j.lfs.2005.12.007 16413584

[14] Davis JM Murphy EA Carmichael MD Zielinski MR Groschwitz CM Brown AS Curcumin effects on inflammation and performance recovery following eccentric exercise-induced muscle damage. *Am J Physiol Regulat Integr Comp Physiol.* (2007) 292:R2168–73. 10.1152/ajpregu.00858.2006 17332159

[15] Huang MT Lysz T Ferraro T Abidi TF Laskin JD Conney AH. Inhibitory effects of curcumin on in vitro lipoxygenase and cyclooxygenase activities in mouse epidermis. *Cancer Res.* (1991) 51:813–9. 1899046

[16] Chih-Li L Jen-Kun L. Curcumin: a potential cancer chemopreventive agent through suppressing NF-κB signaling. *J Cancer Mol.* (2008) 4:11–6.

[17] Tossetta G Fantone S Busilacchi EM Di Simone N Giannubilo SR Scambia G Modulation of matrix metalloproteases by ciliary neurotrophic factor in human placental development. *Cell Tissue Res.* (2022) 390:113–29. 10.1007/s00441-022-03658-1 35794391 PMC9525382

[18] Perugini J Di Mercurio E Tossetta G Severi I Monaco F Reguzzoni M Biological effects of ciliary neurotrophic factor on hMADS adipocytes. *Front Endocrinol.* (2019) 10:768. 10.3389/fendo.2019.00768 31781039 PMC6861295

[19] Huang W-C Chiu W-C Chuang H-L Tang D-W Lee Z-M Wei L Effect of curcumin supplementation on physiological fatigue and physical performance in mice. *Nutrients.* (2015) 7:905–21. 10.3390/nu7020905 25647661 PMC4344567

[20] Fang W Nasir Y. The effect of curcumin supplementation on recovery following exercise-induced muscle damage and delayed-onset muscle soreness: a systematic review and meta-analysis of randomized controlled trials. *Phytother Res.* (2021) 35:1768–81. 10.1002/ptr.6912 33174301

[21] Suhett LG de Miranda Monteiro Santos R Silveira BKS Leal ACG de Brito ADM de Novaes JF Effects of curcumin supplementation on sport and physical exercise: a systematic review. *Crit Rev Food Sci Nutr.* (2021) 61:946–58. 10.1080/10408398.2020.1749025 32282223

[22] Jäger R Purpura M Kerksick CM. Eight weeks of a high dose of curcumin supplementation may attenuate performance decrements following muscle-damaging exercise. *Nutrients.* (2019) 11:1692. 10.3390/nu11071692 31340534 PMC6683062

[23] Ms SAB Waldman DH Krings DB Lamberth DJ Smith DJ McAllister DM. Effect of curcumin supplementation on exercise-induced oxidative stress, inflammation, muscle damage, and muscle soreness. *J Diet Suppl.* (2020) 17:401–14. 10.1080/19390211.2019.1604604 31025894

[24] Nakhostin-Roohi B Nasirvand Moradlou A Mahmoodi Hamidabad S Ghanivand B. The effect of curcumin supplementation on selected markers of delayed onset muscle soreness (DOMS). *Ann Appl Sport Sci.* (2016) 4:25–31. 10.18869/acadpub.aassjournal.4.2.25 30186993

[25] Tanabe Y Chino K Ohnishi T Ozawa H Sagayama H Maeda S Effects of oral curcumin ingested before or after eccentric exercise on markers of muscle damage and inflammation. *Scand J Med Sci Sports.* (2019) 29:524–34. 10.1111/sms.13373 30566760

[26] Tanabe Y Maeda S Akazawa N Zempo-Miyaki A Choi Y Ra S-G Attenuation of indirect markers of eccentric exercise-induced muscle damage by curcumin. *Eur J Appl Physiol.* (2015) 115:1949–57. 10.1007/s00421-015-3170-4 25921600 PMC4536282

[27] McFarlin BK Venable AS Henning AL Sampson JNB Pennel K Vingren JL Reduced inflammatory and muscle damage biomarkers following oral supplementation with bioavailable curcumin. *BBA Clin.* (2016) 5:72–8. 10.1016/j.bbacli.2016.02.003 27051592 PMC4802396

[28] Hsu WH Hsu WB Fan CH Hsu RW. Predicting osteoporosis with body compositions in postmenopausal women: a non-invasive method. *J Orthop Surg Res.* (2021) 16:215. 10.1186/s13018-021-02351-3 33761975 PMC7989015

[29] Hsu WH Chen CL Kuo LT Fan CH Lee MS Hsu RW. The relationship between health-related fitness and quality of life in postmenopausal women from Southern Taiwan. *Clin Intervent Aging.* (2014) 9:1573–9. 10.2147/CIA.S66310 25258526 PMC4172032

[30] Lin MJ Nosaka K Ho CC Chen HL Tseng KW Ratel S Influence of maturation status on eccentric exercise-induced muscle damage and the repeated bout effect in females. *Front Physiol.* (2017) 8:1118. 10.3389/fphys.2017.01118 29354073 PMC5760894

[31] Dias KA da Conceição AR Oliveira LA Pereira SMS Paes SDS Monte LF Effects of curcumin supplementation on inflammatory markers, muscle damage, and sports performance during acute physical exercise in sedentary individuals. *Oxid Med Cell Longev.* (2021) 2021:9264639. 10.1155/2021/9264639 34659641 PMC8516555

[32] Nicol LM Rowlands DS Fazakerly R Kellett J. Curcumin supplementation likely attenuates delayed onset muscle soreness (DOMS). *Eur J Appl Physiol.* (2015) 115:1769–77. 10.1007/s00421-015-3152-6 25795285

[33] Roohi BN Moradlou AN Bolboli L. Influence of curcumin supplementation on exercise-induced oxidative stress. *Asian J Sports Med.* (2017) 8:1–6. 10.5812/asjsm.35776

[34] Takahashi M Suzuki K Kim H Otsuka Y Imaizumi A Miyashita M Effects of curcumin supplementation on exercise-induced oxidative stress in humans. *Int J Sports Med.* (2014) 35:469–75. 10.1055/s-0033-1357185 24165958

[35] Campbell MS Carlini NA Fleenor BS. Influence of curcumin on performance and post-exercise recovery. *Crit Rev Food Sci Nutr.* (2021) 61:1152–62. 10.1080/10408398.2020.1754754 32319320

[36] Haddad M Stylianides G Djaoui L Dellal A Chamari K. Session-RPE method for training load monitoring: validity, ecological usefulness, and influencing factors. *Front Neurosci.* (2017) 11:612. 10.3389/fnins.2017.00612 29163016 PMC5673663

