# Supplementary material for: 12-week curcumin supplementation may relieve postexercise muscle fatigue in adolescent athletes
Source: Front Nutr. 2023 Jan 4;9:1078108. doi: 10.3389/fnut.2022.1078108 (PMC9846492; doi:10.3389/fnut.2022.1078108)
Supplement: Supplementary file 1 [file Data_Sheet_1.PDF]

Supplementary

Supplementary Table 1. Demographics and body composition data of the participants by gender

	Control (N=15)				Curcumin (N=13)			
	Female (N=2) Mean ±SD		Male (N=13) Mean ±SD		Female (N=5) Mean ±SD		Male (N=8) Mean ±SD	
Age (years)	17 ±1		17 ±1		16 ±0		17 ±1	
Height (cm)	159 ±2		169 ±5 ‡		159 ±6		169 ±5 ‡	
W: S: ST	1:1:0		2:8:3		0:5:0		2:4:2	
	Baseline Mean ±SD	12 weeks follow up Mean ±SD	Baseline Mean ±SD	12 weeks follow up Mean ±SD	Baseline Mean ±SD	12 weeks follow up Mean ±SD	Baseline Mean ±SD	12 weeks follow up Mean ±SD
Weight (kg)	53 ±6	55 ±6	62 ±9	62 ±7	58 ±6	58 ±5	64 ±7	65 ±7
BMI (kg/m2)	21 ±2	22 ±3	22 ±2	22 ±3	23 ±3	22 ±3	23 ±2	23 ±2
Body fat mass (kg)	13 ±1	17 ±6	9 ±6	8 ±4	16 ±6 ‡	15 ±4 ‡	10 ±5	10 ±3
Fat free mass (kg)	40 ±5 ‡	38 ±0 ‡	53 ±5	54 ±5	42 ±2 ‡	43 ±3 ‡	54 ±5	55 ±5 †
BMR (kcal)	1237 ±86 ‡	1196 ±11 ‡	1515 ±109	1529 ±107	1276 ±48 ‡	1303 ±58 ‡†	1532 ±100	1564 ±111 †

† $p < 0.05$ between baseline and 12 weeks. ‡ $p < 0.05$ between female and male.

Abbreviation: BMI, body mass index; BMR, basal metabolic rate; F, female; M, male; S, soccer; ST, soft tennis; W, wrestling

Supplementary Table 2. Inflammatory markers, fitness, and muscle fatigue at baseline and the end of study by gender (12 week).

	Control (N=15)				Curcumin (N=13)			
	Female (N=2)		Male (N=13)		Female (N=5)		Male (N=8)	
	Baseline Mean ±SD	12 weeks follow up Mean ±SD	Baseline Mean ±SD	12 weeks follow up Mean ±SD	Baseline Mean ±SD	12 weeks follow up Mean ±SD	Baseline Mean ±SD	12 weeks follow up Mean ±SD
**Inflammatory markers**								
CK (U/L)	364 ±75	552 ±581	459 ±402	533 ±703	279 ±126	209 ±105	275 ±114	340 ±180
MDA (μmol/g CRE)	1.80 ±0.48	2.20 ±0.52	1.42 ±0.58	1.70 ±0.82	1.46 ±0.96	1.17 ±1.59	1.69 ±1.86	1.27 ±0.55
8-OHdG (ng/mg CRE)	2.55 ±0.21 ‡	2.80 ±0.08	4.34 ±1.14	4.43 ±1.56	4.51 ±2.05	3.26 ±0.76	4.96 ±1.50	4.24 ±1.18
TNF-α (pg/ml)	5.27 ±0.71	7.33 ±0.8	7.53 ±1.74	6.65 ±1.73	6.14 ±0.93	6.63 ±1.05	7.12 ±1.50	7.12 ±1.02
**Fitness**								
Sit up (times)	20 ±3	14 ±6	25 ±5	24 ±4	19 ±2	21 ±1	22 ±2	23 ±2
Back strength (kg)	79 ±15 ‡	77 ±14 ‡	114 ±21	126 ±24 †	88 ±11 ‡	110 ±10 ‡	122 ±16	131 ±25
Grip strength (kg)	29 ±3 ‡	31 ± 4‡	40 ±7	41 ±6	28 ±1 ‡	28 ±3 ‡	44 ±8	43 ±6
Vertical jump (cm)	26 ±1 ‡	27 ± 4‡	45 ±7	48 ±6 †	30 ±9 ‡	34 ±4 ‡	44 ±7	46 ±6
Side step (times)	31 ±3	34 ±3	35 ±7	36 ±8	34 ±8	34 ±5	39 ±8	34 ±7
Reaction time (ms)	231 ±30	190 ±32	265 ±61	311 ±63	260 ±27	272 ±39	375 ±181	246 ±57 *†
Close eyes foot balance (s)	18 ±1	22 ±2	54 ±42	61 ±54	57 ±40	55 ±44	124 ±150	82 ±62
Sitting trunk flexion (cm)	15 ±3	16 ±4	17 ±6	16 ±7	19 ±2	20 ±3	14 ±9	12 ±6
Trunk extension (cm)	47 ±3	48 ±4	46 ±5	49 ±6	50 ±6	49 ±2	47 ±7	51 ±8
**Muscle fatigue**								
Muscle fatigue score	6 ±2	7 ±2	7 ±1	7 ±1	7 ±1	4 ±3	6 ±2*	4 ±2*†
Muscle soreness score	6 ±2	7 ±2	8 ±1	7 ±2	8 ±1 ‡	5 ±3 †	6 ±2	4 ±2*†

* $p < 0.05$ between the control and curcumin groups. † $p < 0.05$ between baseline and 12 weeks. ‡ $p < 0.05$ between female and male.

Abbreviation: 8-OHdG, 8-hydroxy-2 deoxyguanosine; CK, creatine kinase; CRE, creatinine; MDA, malondialdehyde; TNF-α, tumor necrosis factor-α; SD, standard deviation.

Supplementary Table 3. Percentage change of body composition, inflammatory markers, fitness, and muscle fatigue between baseline and the end of study by gender (12 weeks)

	Control (N=15)		Curcumin (N=13)	
	Female (N=2)	Male (N=13)	Female (N=5)	Male (N=8)
	Mean ±SD	Mean ±SD	Mean ±SD	Mean ±SD
**Weight and body composition**				
Weight	3 ±1	1 ±4	1 ±6	2 ±3
Body fat mass	25 ±35	5 ±26	-4 ±13	4 ±20
Fat-free mass	-4 ±12	1 ±3	3 ±2	3 ±2
BMR	-3 ±8	1 ±2	2 ±1	2 ±1
**Inflammatory markers**				
CK	38 ±131	16 ±106	-22 ±29	28 ±54
MDA	22 ±50	36 ±90	7 ±168	38 ±163
8-OHdG	10 ±12	2 ±23	-20 ±26	-11 ±28
TNF-α	27 ±22	-9 ±25	10 ±22	4 ±25
**Fitness**				
Sit up	-36 ±17	1 ±18	9 ±16	5 ±16
Back strength	13 ±16	12 ±15	26 ±18	7 ±11
Grip strength	13 ±17	3 ±14	0 ±7	1 ±16
Vertical jump	31 ±28	7 ±9	20 ±27	5 ±8
Side step	17 ±16	5 ±22	0 ±13	-7 ±29
Reaction time	-10 ±40	23 ±39	5 ±16 ‡	-26 ±23 *
Close eyes foot balance	22 ±80	94 ±227	21 ±122	1 ±78
Sitting trunk flexion	-11 ±33	-6 ±25	4 ±18	7 ±47
Trunk extension	2 ±15	6 ±13	-1 ±12	10 ±14
**Muscle fatigue**				
Muscle fatigue score	20 ±8	-9 ±28	-43 ±27	-31 ±32 *
Muscle soreness score	20 ±8	-1 ±22	-36 ±33	-25 ±27

* $p < 0.05$ between the control and curcumin groups. ‡ $p < 0.05$ between female and male
